# Human Zika infection induces a reduction of IFN-γ producing CD4 T-cells and a parallel expansion of effector Vδ2 T-cells

**DOI:** 10.1038/s41598-017-06536-x

**Published:** 2017-07-24

**Authors:** Eleonora Cimini, Concetta Castilletti, Alessandra Sacchi, Rita Casetti, Veronica Bordoni, Antonella Romanelli, Federica Turchi, Federico Martini, Nicola Tumino, Emanuele Nicastri, Angela Corpolongo, Antonino Di Caro, Gary Kobinger, Alimuddin Zumla, Maria Rosaria Capobianchi, Giuseppe Ippolito, Chiara Agrati

**Affiliations:** 1 Cellular Immunology Laboratory, National Institute for Infectious Diseases “Lazzaro Spallanzani”-IRCCS, Rome, Italy; 2 Virology Laboratory, National Institute for Infectious Diseases “Lazzaro Spallanzani”-IRCCS, Rome, Italy; 3 Clinical Department, National Institute for Infectious Diseases “Lazzaro Spallanzani”-IRCCS, Rome, Italy; 4 Department of Microbiology, Immunology and Infectious Diseases, Faculty of Medicine, Université Laval, Quebec, Canada; 5 Division of Infection and Immunity, University College London, and NIHR Biomedical Research centre, University College London Hospitals NHS Foundation Trust, London, United Kingdom; 6 Scientific Direction, National Institute for Infectious Diseases “Lazzaro Spallanzani”-IRCCS, Rome, Italy

## Abstract

The definition of the immunological response to Zika (ZIKV) infection in humans represents a key issue to identify protective profile useful for vaccine development and for pathogenesis studies. No data are available on the cellular immune response in the acute phase of human ZIKV infection, and its role in the protection and/or pathogenesis needs to be clarified. We studied and compared the phenotype and functionality of T-cells in patients with acute ZIKV and Dengue viral (DENV) infections. A significant activation of T-cells was observed during both ZIKV and DENV infections. ZIKV infection was characterized by a CD4 T cell differentiation toward effector cells and by a lower frequency of IFN-γ producing CD4 T cells. Moreover, a substantial expansion of CD3^+^CD4^−^CD8^−^ T-cell subset expressing Vδ2 TCR was specifically observed in ZIKV patients. Vδ2 T cells presented a terminally differentiated profile, expressed granzyme B and maintained their ability to produce IFN-γ. These findings provide new knowledge on the immune response profile during self-limited infection that may help in vaccine efficacy definition, and in identifying possible immuno-pathogenetic mechanisms of severe infection.

## Introduction

Zika virus (ZIKV) is an emerging arbovirus of the Flaviviridae family isolated in Uganda in 1947^1^ and usually causes a mild and self-limiting infection. Nevertheless, several data strongly indicate a high rate of primary microcephaly and Guillain-Barré syndrome during ZIKV infection in French Polynesia and Brazil^2–5^. There are currently no licensed medical interventions (drugs, other therapeutics or vaccines) available to treat or prevent ZIKV infection and the development of severe disease. The wide cross reactivity among different flavivirus^6^ and the risk associated with the antibody dependent enhancement^7^ strongly request the identification of protective and pathogenetic immune signature to ZIKV.

ZIKV infects Dendritic cells (DC)^8^ and antagonizes Type I Interferon Responses, thus subverting DC immunogenicity^9^. During the acute and convalescent phases of ZIKV infection, increased levels of Th1, Th2, Th9, Th17 cytokines have been reported, suggesting that a polyfunctional T-cell response is required for recovery from ZIKV infection^10^. Nevertheless, an unbalanced immunoactivation with high levels of IL-6 and IL-8 in the cerebrospinal fluid has been associated to encephalomyelitis^11^. Although the main role played by humoral response^12^, an involvement of CD8 T cell response in the protection against ZIKV infection has been recently suggested in a mouse model of ZIKV infection^13^. Moreover, the activation of T-cells and their Th1 polarization has been also recently demonstrated in mice^14^. No data are available on the cellular-mediated immune response during the acute phase of ZIKV infection in humans.

The aim of this study was to study and compare the phenotype and functionality of T-cells in patients with acute ZIKV and Dengue viral (DENV) infections.

## Results

### ZIKV infection expanded CD8 and DN T cells and induced T-cell activation

**Figure 1:**
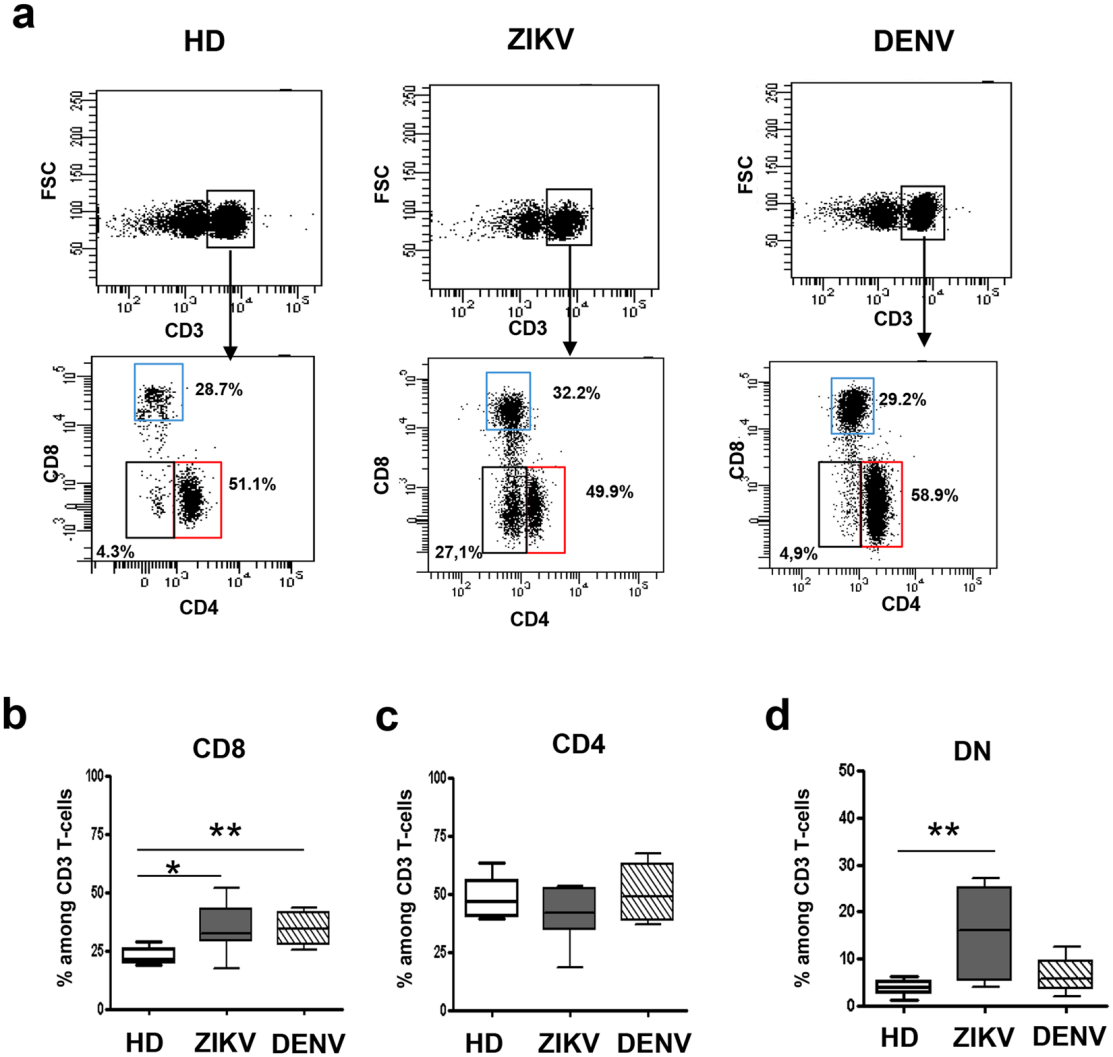
T-cell subsets during ZIKV infection. Flow cytometric panels of CD3, CD8, CD4 and DN T-cells in one representative ZIKV-infected, one DENV-infected patient and in one representative HD (**a**). The frequency of CD8 (**b**), CD4 (**c**) and DN (**d**) T-cells was compared in HD (white bars), ZIKV (grey bars) and in DENV (hatched bars). *p < 0.05, **p < 0.01.

The characterization of T-cell subsets in healthy donors (HD), ZIKV and DENV-infected patients were performed by multiparametric flow cytometry (Fig. 1). Representative panels from one HD, one ZIKV- and one DENV-infected patient are shown in Fig. 1a. The CD8 T-cells frequency was different in the three groups (Kruskal Wallis, KW < 0.05). In particular, when compared to HD, a significant higher CD8 T-cell frequency was observed both in ZIKV- and in DENV-infected patients (Fig. 1b). In contrast, no difference in CD4 T-cell frequency was observed among groups (Fig. 1c). Finally, the frequency of CD3^+^CD4^−^CD8^−^ T-cell population (double negative, DN T-cells) was different in the three groups (KW < 0.05). Specifically, a significant expansion of DN T-cells was observed during ZIKV infection (Fig. 1d).

**Figure 2:**
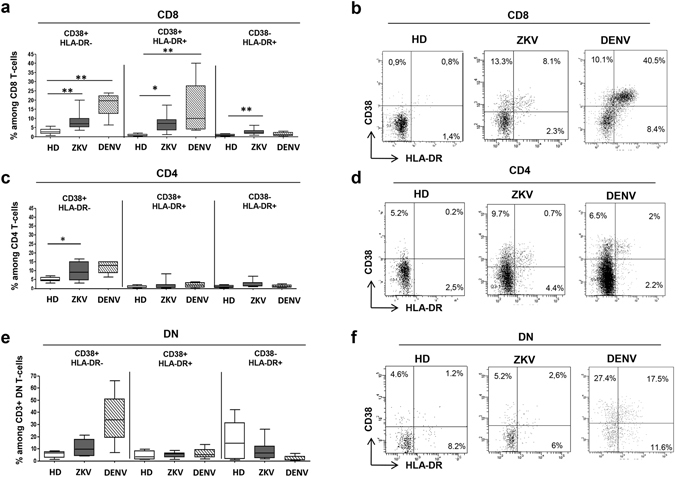
Activation of CD8, CD4 and DN T-cells during ZIKV infection. The frequency of activated cells (CD38^+^, CD38^+^HLADR^+^, HLADR^+^) within CD8^+^ (**a**), CD4^+^ (**b**) and DN (**c**) T-cells was analyzed by flow cytometry in HD (white bars), ZIKV (grey bars) and in DENV (hatched bars). *p < 0.05; **p < 0.01.

The impact of ZIKV infection on T-cell activation was evaluated by analyzing the expression of activation markers CD38 and HLA-DR on CD8 (Fig. 2a,b), CD4 (Fig. 2c,d) and on DN (Fig. 2e,f) T-cells. A significant difference among HD, ZIKV and DENV was observed in the activation profile of CD8 (KW < 0.05) and of CD4 T cells (KW < 0.05). Specifically, when compared to HD, a higher frequency of CD38^pos^ and of CD38^pos^/HLA-DR^pos^ CD8 T-cells was observed both in ZIKV and in DENV patients (Fig. 2a). Moreover, a significant higher frequency of CD38^neg^/HLA-DR^pos^ CD8 T-cells was observed in ZIKV-patients than in HD (Fig. 2a). CD4 T-cells showed a lower level of activation than CD8 T-cells both in ZIKV and DENV patients but CD38^pos^ CD4 T-cells were significantly higher in ZIKV and DENV than in HD (Fig. 2c). Although a trend of CD38 increase on DN T cells during DENV infection, no significant difference was observed on the activation of DN T-cells among HD, ZIKV and DENV, probably due to the small sample size (Fig. 2e).

### ZIKV infection induced T-cell differentiation

**Figure 3:**
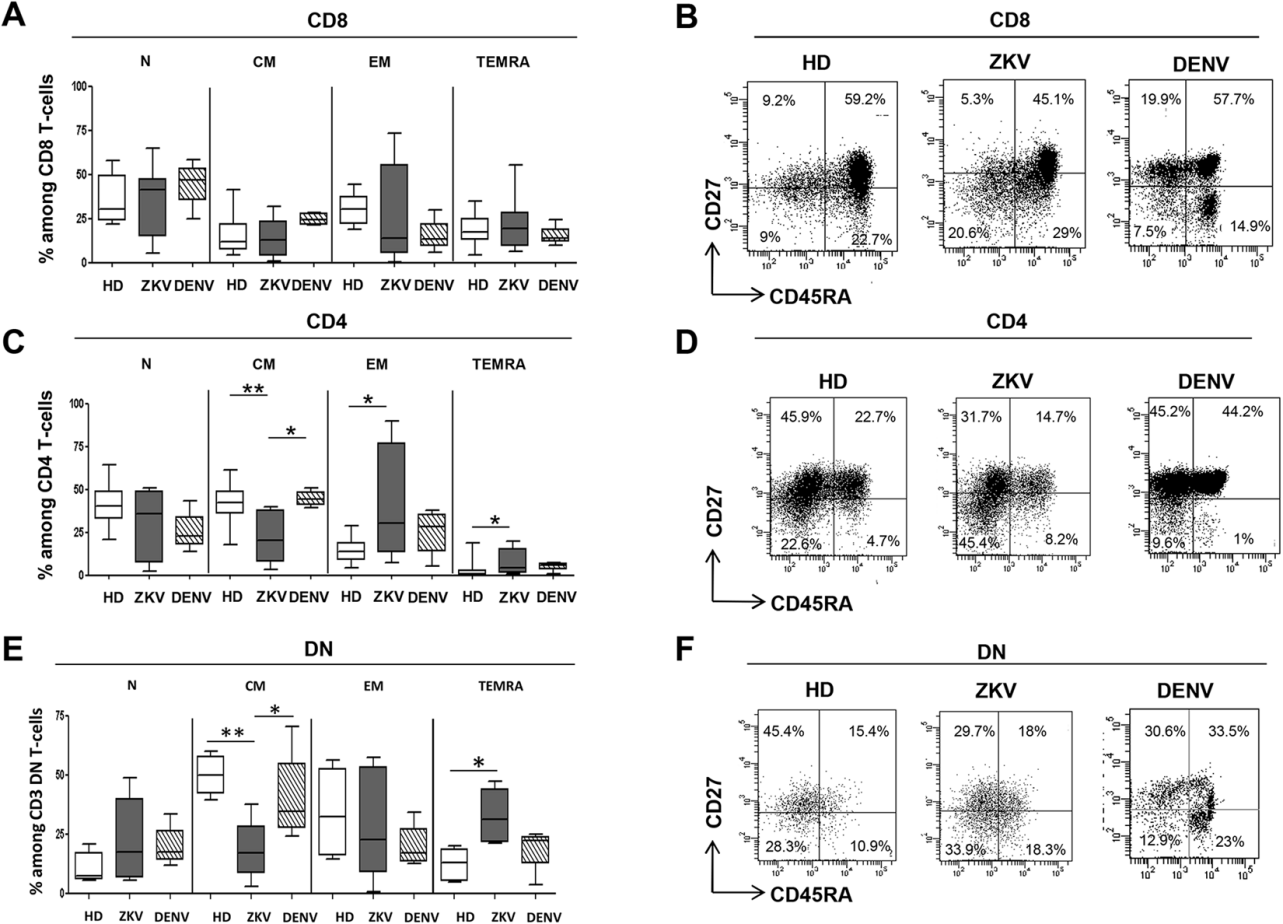
Differentiation profile of CD8, CD4 and DN T-cells during ZIKV infection. The frequency of naive (N, CD45RA^pos^CD27^pos^), central memory (CM, CD45RA^neg^CD27^pos^), effector memory (EM, CD45RA^neg^CD27^neg^) and terminally differentiated (TEMRA, CD45RA^pos^CD27^neg^) CD8^+^ (**a**), CD4^+^ (**b**) and DN (**c**) T-cells was analyzed by flow cytometry in HD (white bars), ZIKV (grey bars) and in DENV (hatched bars). *p < 0.05; **p < 0.01.

The analysis of the differentiation profile was assessed by monitoring the expression of CD45RA and CD27 on CD8 (Fig. 3a,b), CD4 (Fig. 3c,d) and on DN (Fig. 3e,f) T-cells allowing to discriminate subsets of naive (N: CD45RA^pos^CD27^pos^), central memory (CM: CD45RA^neg^CD27^pos^), effector memory (EM: CD45RA^neg^CD27^neg^) and terminally differentiated (TEMRA: CD45RA^pos^CD27^neg^) T-cells. The analysis of CD8 T-cells did not show any difference on the differentiation profile between ZIKV, DENV and HD (Fig. 3a). In contrast, a significant difference was observed in the differentiation profile of CD4 (KW < 0.05) and DN (KW < 0.05) T-cells. In particular, ZIKV patients showed a lower frequency of CM-CD4 T-cells in comparison to both HD and DENV patients, and a parallel higher frequency of EM-CD4 T-cells and TEMRA-CD4 T-cells in ZIKV respect to HD (Fig. 3c). Similar results were obtained by analysing DN T-cells: indeed, a lower frequency of CM-DN T- cells was observed in ZIKV in comparison to both HD and DENV patients and a parallel higher frequency of TEMRA-DN T-cells in ZIKV respect to HD (Fig. 3e). Finally, an inverse correlation between days after symptoms onset and EM CD8 T-cell frequency was observed (Pearson R: −0.76, R Squared 0.58, p = 0.04).

### ZIKV infection induced the expansion of DN T-cell expressing Vδ2 TCR

**Figure 4:**
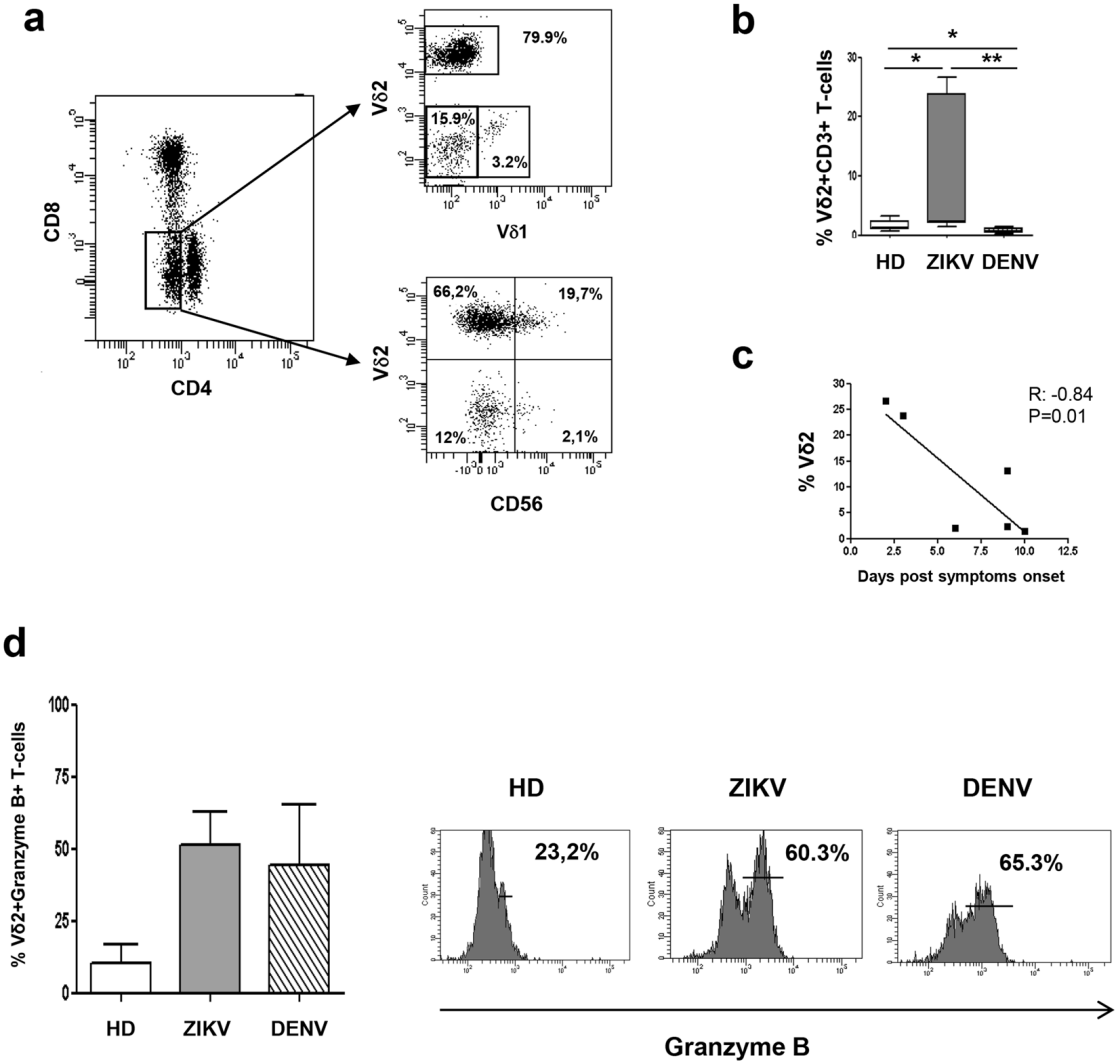
The expanded DN T-cells express Vδ2 TCR. Flow cytometric panels showing the expression of Vδ1-TCR, Vδ2 TCR and CD56 marker on DN T-cells are shown (**a**). The frequency of Vδ2 within CD3 T-cells (**b**) was compared in HD (white bars), ZIKV (grey bars) and in DENV (hatched bars). The correlation between time after sympthoms onset (days) and the Vδ2 T cell frequency (**c**) during Zika infection is shown. The frequency of granzyme positive Vδ2 T cells (**d**) was compared in HD (white bars), ZIKV (grey bars) and in DENV (hatched bars). *p < 0.05.

To further characterize the phenotype of expanded DN T-cells observed during ZIKV infection, the expression of the γδ TCR (Vδ1 and Vδ2) and CD56 NK marker was analysed by flow cytometry. Results showed that the large majority of DN T-cells expressed a Vδ2 TCR (Fig. 4a), suggesting that the DN T-cell population expanded during ZIKV infection belongs to the Vδ2 T-cell subset. Accordingly, the frequency of Vδ2 T-cells within CD3 T-cells was significantly different among HD, ZIKV and DENV (KW < 0.05). In particular, Vδ2 T-cells was significantly higher in ZIKV patients than in HD and in DENV patients (Fig. 4b). Notably, the Vδ2 T-cell expansion was more pronounced in patients sampled during the first 2-3 days from symptoms onset [Pt1 and Pt2: Range (23.8–26.6%)] than in the other patients sampled after 6 days [Pt3-6: Range (1.5–13.1%)], and an inverse correlation between days after symptoms onset and Vδ2 T-cell frequency was observed (Pearson R: −0.84, R Squared 0.70, p = 0.01, Fig. 4c). Although not reaching the statistical significance, the analysis of granzyme expression showed a trend of increase of Granzyme B positive Vδ2 T-cells in ZIKV patients compared to HD (KW = 0.07, Fig. 4d).

### ZIKV infection induced a reduction in IFN-γ production by T-cells

**Figure 5:**
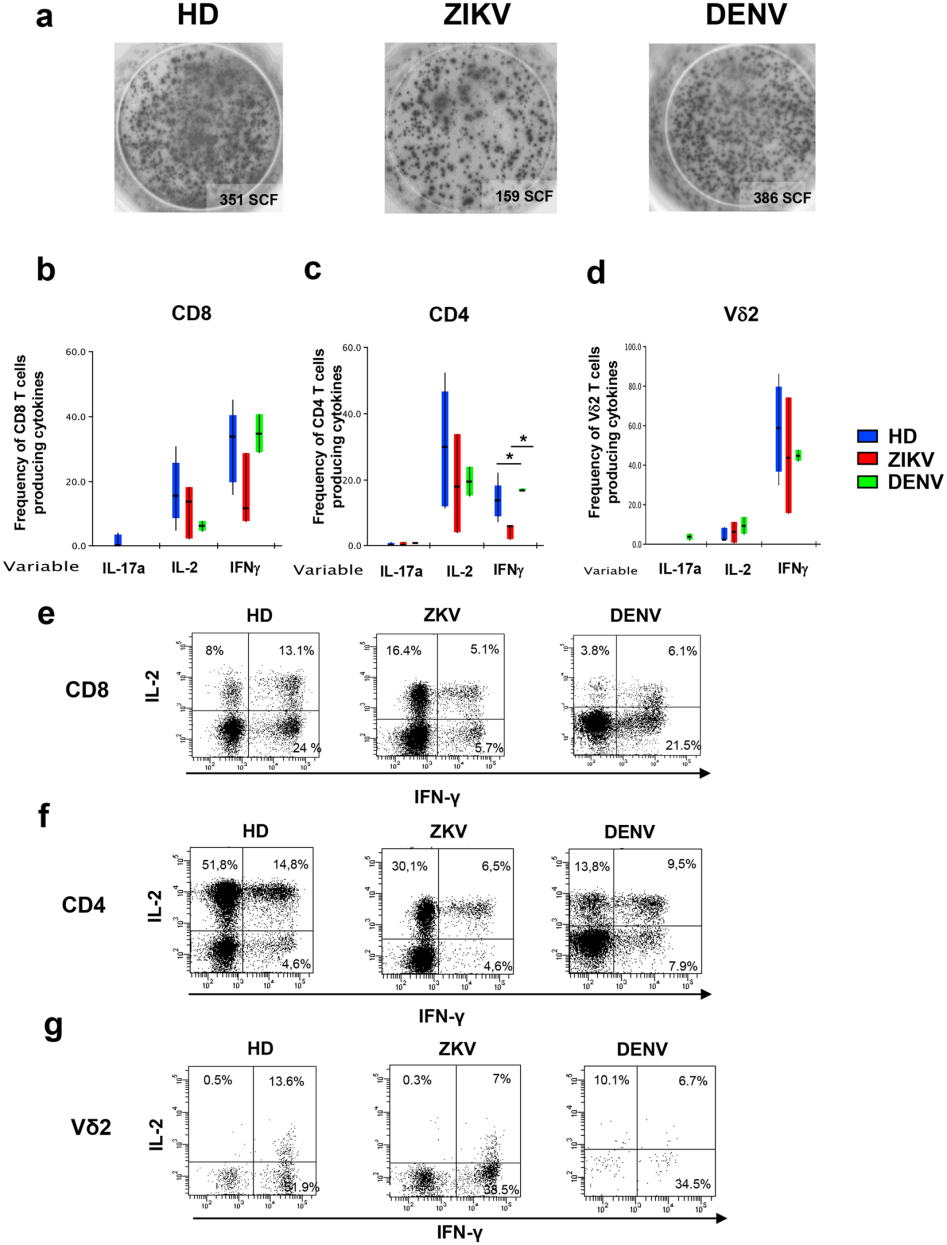
Functional profile of CD8 and CD4 T-cells response to ZIKV infection. IFN-γ production by T-cells after PHA stimulation was analyzed by EliSpot assay (**a**). The frequency of CD8 (**b**), CD4 (**c**) and Vδ2 (**d**) T-cells producing IL-17A, IL-2 and IFN-γ after PMA/Ionomycin stimulation is shown in ZIKV (red bars), DENV (green bars) and HD (blue bars). *p < 0.05.

In order to define the functional properties of αβ and γδ T-cells during ZIKV infection, we tested their ability to produce cytokines by EliSpot assay (Fig. 5a) and flow cytometry (Fig. 5b,e) after mytogenic stimulation. Representative cytometric panels are shown (Fig. 5e). As shown in Fig. 5a, a significant reduction in the frequency of IFN-γ producing T-cells was observed in ZIKV patients than in HD [ZIKV: median 593 SFC/10^6^PBMC (IQR: 517–620) vs. HD: median 1165 SFC/10^6^PBMC (IQR: 950–1180), p < 0.05]. We then analysed three different cytokines by flow cytometry, focusing on those that were found elevated in acute ZIKV patients^10^. The analysis of IFN-γ-producing CD4 T-cells revealed a significant difference among HD, ZIKV and DENV (KW < 0.05). In particular, ZIKV infection was associated to a reduction of IFN-γ production by CD4 T-cells (Fig. 5c). Of note, Vδ2 T-cells maintained their ability to produce IFN-γ as well as HD and DENV (Fig. 5d). No significant differences were observed in IL-17A and IL-2 producing CD4, CD8 and Vδ2 T-cells.

## Discussion

The definition of the immunological response to ZIKV infection in humans represents a key issue to identify a protective profile useful for vaccine development and for pathogenesis studies. Our study is the first to report the dynamics of T-cell profile and function in patients with acute ZIKV infection. There are 4 important findings from our study: 1) activation of both CD4 and CD8 T-cells; 2) CD4 T cell differentiation toward effector cells 3) substantial expansion of effector Vδ2 T-cells in the first days after symptoms onset 4) cytokine modulation in CD4 T-cells with a reduction of IFN-γ production.

Activation of CD8 and CD4 T-cells has been extensively described in several viral infections and likely represents the efforts of the immune system to counteract viral replication. Nevertheless, an excessive T-cell activation observed during severe viral diseases such as severe DENV^15^ or Ebola viral (EBOV) infection^16–18^ may be harmful to the host, by increasing inflammation and promoting anergy of protective T-cells. We found that ZIKV infection induced a significant activation of CD8 and CD4 T-cells, confirming recent data obtained in mice^14^. The frequency of CD8 T-cells co-expressing both CD38 and HLA-DR was similar in ZIKV and in DENV patients^15^, but was lower than during EBOV infection^16, 18^, suggesting that a moderate activation may be associated to a protective activity and to a mild profile of the disease. In acute infection such as of DENV^19^ or Ebola virus^16–18^, the activation of T-cells is associated to an increased expression of apoptotic markers. The role of apoptosis during ZIKV infection remains to be defined.

The ability of T-cells to differentiate into effectors is a key feature of a protective response. During ZIKV infection, a CD4 T-cells differentiation towards effectors was described, suggesting the induction of a well coordinate immune response. In particular, CD4 T-cells differentiated in effector memory and terminally differentiated cells, suggesting the acquisition of a cytokine-producing and cytotoxic profiles. Other study are necessary to deeply explore the functional properties of effector CD4 T-cells. An effector phenotype of T-cells was also described in the mouse model of ZIKV infection^14^. Nevertheless, although the expression of an effector phenotype, a significant reduction of IFN-γ producing CD4 T-cells was observed respect to both HD and DENV patients. Was can speculate that CD4 T-cells during Zika infection were polarized to produce cytokine and chemokines other than IFN-γ. The ability of ZIKV to inhibit Type-I IFNs production and response was reported^9, 20^ but the reduction of IFN-γ production by T-cells represent a new finding whose role in the context of protection/pathogenesis needs further investigations. No differences in the frequency of IL-2 and IL-17-producing CD4 and CD8 T-cells was observed, suggesting that ZIKV did not modify the ability of T-cells to produce these cytokines that indeed were found higher in the sera of ZIKV patients^10^.

The current study reports for the first time a substantial expansion of Vδ2 T-cells during ZIKV infection, that was more pronounced during the first days from symptoms onset. Expanded Vδ2 T-cells presented an effector phenotype and expressed granzyme B. A pillar role of Vδ2 T-cells in a well orchestrated immune response to viral infection is well documented^21^. Indeed, human γδ T-cells may affect the progression and outcome of infectious diseases^22, 23^. Activated Vδ2 T-cells are able to exert direct antiviral activities and to perform several stimulatory activities on both innate and adaptive immune cells^24–27^. Several reports suggest a protective role of γδ T-cells during other acute viral infections^22, 23, 28–30^. γδ T-cells expand quickly in response to WNV infection, produce significant amount of IFN-γ, limiting the viral load and protecting the host from lethal encephalitis^29, 30^. We could not directly correlate the Vδ2 T-cell expansion with a protective effect during ZIKV infection, since all the patients included in this study showed a mild clinical course of the disease and no sequelae were observed. A direct comparison between severe and mild diseases may be helpful in supporting a protective role of γδ T-cells during ZIKV infection. The analysis of Vδ2 T-cells may also be important to better understand the pathogenesis associated with severe clinical complications of ZIKV infection such as microcephaly in foetuses^2, 3^ and/or Guillain Barré syndrome in adults^31^. Interestingly, an expansion of Vδ2 T-cells has been associated with recurrent abortions^32, 33^. Furthermore, the Th1 profile of expanded Vδ2 T-cells might contribute to placenta damage and/or inflammation that has been correlated with foetal brain damage during ZIKV infection^34^. Finally, γδ T-cells may play a role during autoimmune diseases^35^ and in particular during Guillain Barré syndrome^36–38^, contributing to autoimmune damage.

In summary, this study shows for the first time the activation of both CD8 and CD4 T-cell subsets, a reduction of IFN-γ producing CD4 T-cells and a prolific expansion of effector Vδ2 T-cells during acute ZIKV infection. Further studies on a larger sample size (including mild and severe ZIKV-associated diseases) are required to clarify the role of the Vδ2 T-cells and of reduced IFN-γ production by CD4^+^ T-cells in the protection and/or pathogenesis of ZIKV infection and to help develop rational strategies for new vaccine and therapeutics.

## Materials and Methods

### Ethical Approval

The study was approved by the National Institute for infectious Diseases (INMI), L Spallanzani of Rome Ethics Committee (approval number: 14/2015) and all patients gave written informed consent. All methods were performed in accordance with the relevant guidelines and regulations.

### Patients enrolled

Patients returning from travel in Latin America, in India or Southeast Asia (Table 1) were enrolled at the National Institute for infectious Diseases (INMI), L Spallanzani of Rome (in travel clinic) after diagnosis of ZIKV or DENV infections. Specifically, 7 Italian patients (6 females and 1 male; median age 41.2 years) were positive for ZIKV (both serology and PCR), and 5 Italian patients (2 females and 3 males; median age: 48.5 years) were positive for DENV (both serology and PCR). 10 healthy donors (HD) were enrolled as controls. The main manifestations of ZIKV and DENV are summarized in Table 1. The serological and virological data are reported in Table 2.

**Table 1:** Clinical features of enrolled patients.

Patients (n° pts)	Gender (M/F)	Age	Travel History (n° pts)	Symptoms onset (range, days)	Symptoms
ZIKV (7)	1/6	37 ± 9.7	Brazil (2) Mexico (2) Barbados (3)	2–9	cutaneous rash: 7/7 fever: 4/7 headache: 3/7 arthralgia: 6/7
DENV (5)	2/3	45.2 ± 14.7	Brazil (1) Indonesia (2) Vietnam (1) India (1)	4–9	cutaneous rash: 4/5 fever: 4/5 headache: 0/5 arthralgia: 4/5

**Table 2:** Serological and virological data of ZIKV- and DENV-infected patients.

Patient ID (Gender)	Days after symptoms onset	Sample	ZIKV Real Time RT-PCR^a^	DENV Real Time RT-PCR^b^	CHIKV Real Time RT-PCR^c^	Anti ZIKV IgG^d^	Anti ZIKV IgM^d^	Anti DENV IgG^d^	Anti DENV IgM^d^	Anti CHIKV IgG^d^	Anti CHIV IgM^d^
Pt1 (F)	3	Serum	Negative	Negative	Negative	1:320	<1:20	1:640	<1:20	<1:20	<1:20
		Urine	Positive	Negative	ND	ND	ND	ND	ND	ND	ND
		Saliva	Positive	Negative	ND	ND	ND	ND	ND	ND	ND
Pt2 (F)	2	Serum	Positive	Negative	Negative	<1:20	<1:20	<1:20	<1:20	<1:20	<1:20
		Urine	Positive	Negative	ND	ND	ND	ND	ND	ND	ND
		Saliva	Negative	Negative	ND	ND	ND	ND	ND	ND	ND
Pt3^e^ (F)	9	Serum	Negative	Negative	Negative	1:160	1:80	1:40	<1:20	<1:20	<1:20
		Urine	Positive	Negative	ND	ND	ND	ND	ND	ND	ND
Pt4 (M)	9	Serum	Negative	Negative	Negative	1:80	1:160	1:20	<1:20	<1:20	<1:20
		Urine	Positive	Negative	ND	ND	ND	ND	ND	ND	ND
		Saliva	Negative	Negative	ND	ND	ND	ND	ND	ND	ND
Pt5 (F)	9	Serum	Positive	Negative	Negative	1:160	1:80	1:160	<1:20	<1:20	<1:20
		Urine	Positive	Negative	ND	ND	ND	ND	ND	ND	ND
		Saliva	Negative	Negative	ND	ND	ND	ND	ND	ND	ND
Pt6 (F)	6	Serum	Negative	Negative	Negative	1:20	1:80	<1:20	<1:20	<1:20	<1:20
		Urine	Positive	Negative	ND	ND	ND	ND	ND	ND	ND
		Saliva	Negative	Negative	ND	ND	ND	ND	ND	ND	ND
Pt7 (F)	9	Serum	Negative	Negative	Negative	1:160	1:80	<1:20	<1:20	<1:20	<1:20
		Urine	Positive	Negative	ND	ND	ND	ND	ND	ND	ND
		Saliva	Negative	Negative	ND	ND	ND	ND	ND	ND	ND
Pt8 (M)	9	Serum	Negative	Positive	Negative	<1:20	<1:20	Weak reactivity	1:40	<1:20	<1:20
		Urine	Negative	Positive	ND	ND	ND	ND	ND	ND	ND
		Saliva	Negative	Negative	ND	ND	ND	ND	ND	ND	ND
Pt9 (M)	4	Serum	Negative	Positive	Negative	<1:20	<1:20	<1:20	<1:20	<1:20	<1:20
		Urine	Negative	Negative	ND	ND	ND	ND	ND	ND	ND
		Saliva	Negative	Negative	ND	ND	ND	ND	ND	ND	ND
Pt10 (M)	5	Serum	Negative	Positive	Negative	<1:20	1:40	1:40	1:80	<1:20	<1:20
		Urine	Negative	Positive	ND	ND	ND	ND	ND	ND	ND
		Saliva	Negative	Negative	ND	ND	ND	ND	ND	ND	ND
Pt11 (F)	5	Serum	Negative	Positive	Negative	<1:20	<1:20	<1:20	Weak reactivity	<1:20	<1:20
		Urine	Negative	Positive	ND	ND	ND	ND	ND	ND	ND
		Saliva	Negative	Positive	ND	ND	ND	ND	ND	ND	ND
Pt12 (F)	4	Serum	Negative	Positive	Negative	<1:20	<1:20	1:40	<1:20	<1:20	<1:20
		Urine	Negative	Negative	ND	ND	ND	ND	ND	ND	ND
		Saliva	Negative	Negative	ND	ND	ND	ND	ND	ND	ND

DENV: dengue virus; ZIKV: Zika virus; RT-PCR: Reverse transcription polymerase chain reaction; ND: not done. ^a^ZIKV specific Real Time RT-PCR^39^. ^b^CDC DENV-1–4 Real-Time RT-PCR Assay for Detection and Serotype Identification of Dengue Virus^38^. ^c^CHIKV Real-Time RT-PCR Assay targeting the E1 structural protein^40^. ^d^IgG and IgM indirect immunofluorescence assay Euroimmun Flavivirus Mosaic 2. Reference values (titre): <1:20 = negative; ≥1:20 = positive. ^e^This patient is vaccinated against Yellow Fever.

### Serological and virological assays

Whole-blood samples were collected using sterile EDTA-treated Vacutainer tubes (Becton Dickinson). The ZIKV and DENV diagnosis was performed by using both molecular and serological tests and other arboviral infections (Chikungunya virus) were ruled out. Serum samples were tested by indirect immunofluorescence assay for both IgM and IgG antibodies against ZIKV, DENV and Chikungunya virus (CHIKV) (IFA Arboviral Fever Mosaic 2 IgM and IgG Euroimmun AG, Luebeck, Germany). Serum, saliva and urine samples from patients were tested also by Real Time RT-PCR for ZIKV, DENV and CHIKV RNA^39–41^.

### Leucocytes isolation and flow cytometry

Peripheral Blood Mononuclear Cells (PBMC) were isolated by Ficoll procedure, counted and stored at −80 °C in 90% FCS/10% DMSO (Euroclone). Cryopreserved PBMC were rapidly thawed, washed with PBS 1X and analyzed by flow cytometry using the following anti-human monoclonal antibodies to assess T-cell subsets, and T-cell activation, differentiation and functional profile: CD4 V450 (Clone RPA-T4), CD8 APC H7 (Clone SK1), CD8 Pe Cy 7 (Clone RPA T8), CD3 PerCp Cy 5.5 (Clone SP 34-2), CD3 FITC (Clone UCHT1), CD3 V500 (Clone UCHT1), CD27 APC H7 (Clone M-T271), CD38 APC (Clone HIT2), HLA-DR PerCp (Clone L243), Vδ2 TCR PE (Clone B6), INF-γ PE-Cy 7 (Clone B27), Vδ2 TCR FITC (Clone B6), Granzyme –B AlexaFLUOR647 (Clone GB11), CD45 AmCyan (Clone 2D1), purchased from BD Pharmigen; CD45RA PerCp-Vio 700 (Clone REA 562), IL 2 APC (Clone N7.48 A), purchased from Macs Miltenyi Biotec; TCR Delta TCδ1 FITC (Clone TS-1), purchased from ThermoFisher Scientific; CD56 (Clone N 901), purchased from Beckman Coulter; and finally, IL-17A PE (Clone eBio64CAP17), purchased from Affymetrix eBioscience. Briefly, 5 × 10^5^ PBMC were incubated for 15 min at 4 °C with the indicated mAbs. After washing (PBS/1%BSA/0.1% sodium azide), samples were fixed with 2% paraformaldehyde (Electron Microscopy Sciences) and immediately acquired using a FACSCanto II flow cytometer. The general gating strategy was: FSC-A v. SSC-A > CD3pos > CD4 pos or CD8 pos or CD4 negCD8 neg cells. The activation analysis was performed by analyzing CD38 and HLA-DR expression on i) CD4 pos, ii) CD8 pos and iii) CD4 negCD8 neg cells. The differentiation profile was performed by analyzing CD27 and CD45RA on i) CD4 pos, ii) CD8 pos and iii) CD4 negCD8 neg cells. The gating strategy for the analysis of CD3posCD4 negCD8 neg cells was: FSC-A v. SSC-A > CD3pos > CD4 negCD8 neg cells > Vδ1 or Vδ2 or CD56. The gating strategy for the analysis of Vδ2 T cells was: FSC-A v. SSC-A > CD3pos > Vδ2 T cells > Granzyme B. Positive gates were selected on the basis of isotype matched controls. Results are shown as Box and Whiskers: the box encompasses the interquartile range of individual measurements, the horizontal bar-dividing line indicates the median value, and the whiskers represent maximum and minimum values.

### Elispot assay

T-cell functionality during Zika acute infection was assessed by detecting interferon-gamma (IFN-γ) production using an enzyme-linked immunosorbent spot-forming cell assay (ELISpot) after PHA stimulation. Peripheral blood mononuclear cells (PBMCs) were thawed in culture medium (RPMI 1640, 10% FCS, 2 mmol/liter L-glutamine) and assessed for vitality by Trypan Blue exclusion, counted, and plated at 3 × 10^5^ cells/well in ELISpot plates (AID GmbH, Strabberg, Germany). PBMCs were then stimulated with PHA, included in the Elispot kit, for 24 hours with 5% of CO_2_. At the end of incubation, the ELISpot assay was developed according to manufacturer’s instructions. Spontaneous cytokine production (background) was assessed by incubating PBMC with 1 μg/ml αCD28 and αCD49d (IgG1, clones CD28.2 and 9f10, respectively; Becton Dickinson, Mountain View, CA). Results are expressed as spot forming cells (SFC)/10^6^ PBMCs in stimulating cultures after subtracting spontaneous background.

### Polyfunctional analysis

Cytokines and chemokines expression were simultaneously examined by using multiparametric flow cytometry. Briefly, PBMCs from ZIKV infected patients and from healthy donors were stimulated for 4 hours with PMA/Ionomycin (PMA 50 nM/ml and Ionomycin 1μM/ml, Sigma Aldrich) for CD4 T-cells, and IPH 1101 (3 μM, Innate Pharma, Marseille, FR) for Vδ2 T-cells. Brefeldin A (10 μg/ml, Serva) was added after 1 hour of stimulation. First, surface staining was performed using anti-human CD3, anti-CD4, anti-CD8 and anti- Vδ2 conjugated monoclonal antibodies prepared using PBS/BSA/NAN3 solution. After an incubation of 10 minutes at 4 °C, cells were washed and fixed with 2% paraformaldehyde (Electron Microscopy Sciences) and then stained for intracellular cytokines using anti-human IFN-γ, IL-2, IL-17A conjugated monoclonal antibodies prepared using a solution of PBS/BSA/NAN3 with 0.5% saponin. After an incubation of 20 minutes, cells were washed and acquired on FACSCanto II (BD, Buccinasco, Milano, Italy) flow cytometer and analyzed using Diva (BD) and FlowJo Softwares (TreeStar, Olten, Switzerland). Results are shown as Box and Whiskers: the box encompasses the interquartile range of individual measurements, the horizontal bar-dividing line indicates the median value, and the whiskers represent maximum and minimum values.

### Statistical analyses

Statistical significance was determined by GraphPad Prism software. The differences in the median values among multiple groups (HD, ZIKV and DENV) were evaluated by non-parametric Kruskall-Wallis (KW) test. The differences in the median values between two groups were evaluated by non-parametric Mann-Whitney test. A p–value < 0.05 was considered significant.
